# Causes of low vision and major low-vision devices prescribed in the low-vision clinic of Nepal Eye Hospital, Nepal

**DOI:** 10.1080/19768354.2017.1333040

**Published:** 2017-06-13

**Authors:** Kishor Sapkota, Douk Hoon Kim

**Affiliations:** a Universidade do Minho, Braga, Portugal; b Nepal Eye Hospital, Kathmandu, Nepal; c Department of Optometry, Masan University, Masanhoewon-gu, South Korea

**Keywords:** Cataract, low vision, nystagmus, telescope, vision impairment

## Abstract

Visual impairment is a major public health problem. Identifying the main causes of low vision and the major low-vision devices prescribed will help to develop and implement the low-vision rehabilitation service. We find out the causes of low vision and the low-vision devices prescribed in the low-vision clinic of Nepal Eye Hospital. A retrospective cross-sectional review of all patients attending the low-vision clinic from 1 May 2009 to 31 April 2011. Patients having visual acuity less than 3/60 in the better eye with best refractive correction were excluded. Of the 137 patients, the mean age was 32.53 ± 22.90 years; 71.5% were male and 67.88% were under 40. The major causes of low vision were nystagmus (30.70%), high refractive error (22.62%), cataract (15.30%), retinitis pigmentosa (15.30%) and age-related macular degeneration (13.10%); 78.10% patents were wearing glasses while telescopes were prescribed for 29.20% patients. Nystagmus, high refractive error and cataract are the main causes of low vision in Nepal. The majority of the low-vision patients seen in this clinic are of working age. Telescopes are the major low-vision device prescribed. We review approach the cause of low-vision problem in low-vision clinic Nepal Eye Hospital, Nepal.

## Introduction

Visual impairment is a major public health problem in developing countries. Someone is described as having low vision if the best-corrected visual acuity is less than 6/18 but equal to or better than 3/60 in the better eye. Moreover, someone with a visual field of less than 10 degrees is also defined as having low vision. People with visual acuity poorer than 3/60 are classified as blind (WHO 1992).

Functional limitations caused by poor vision occur at visual acuity levels that are much better than the criterion that defines legal blindness (Katz & Tielsch 1996). Low vision is responsible for a high proportion of social care service use and results in important reductions in functional status.

The World Health Organization (WHO) estimated that 161 million and 37 million, respectively, were visually impaired and blind in 2002 worldwide (Pascolini et al. 2004). Studies on prevalence and causes of visual impairment and blindness in different parts of the world give varied results (Buch et al. 2001; Liang et al. 2008; Cruciani et al. 2011).

In Nepal, it is estimated that 1.85% of the total population is living with low vision (Brilliant et al. 1985). Although some studies have addressed the low vision in different parts of Nepal, relatively few investigations have focused on the causes of low vision and their association with different factors like age, sex and ethnicity (Kansakar et al. 2009). In a survey of blindness of Nepal, the main causes of blindness were cataract, retinal diseases, trachoma, trauma, amblyopia and malnutrition (Brilliant et al. 1985). Similarly, a recent study from Bhaktapur, Nepal showed that major causes of low vision were cataract and refractive error in the adult age group (Thapa et al. 2011).

This study focused on finding the major causes of low vision in patients, referred to a low-vision clinic in Nepal. The associations between these causes and the other factors (such as age) were also identified. The most frequently prescribed optical devices were also evaluated. This study aims to help the development and implementation of low-vision rehabilitation programmes effectively in this part of world.

## Age and gender distribution

One hundred and thirty-seven patients were included in this study. The mean age of the patients was 32.53 ± 22.90 years, with a range of 4–85 years. About two-thirds (71.5%, 98) of the patients were male. The mean age of the male was 34.05 ± 22.85 years while that of female was 28.69 ± 22.89 years. There was significant difference in the numbers of males and females in terms of the age group (Pearsons’ Chi-Square test, *p* < .05) (Table 1).

**Table 1.** Age and gender distribution.

Age group in year	Male	Female	Total
0–20	30	23	53
21–40	37	5	42
41–60	13	5	18
61–80	14	4	18
81–100	4	2	6
Total	98	39	137

Most of the low-vision patients seen in this clinic were of working age. This will have consequences for the health and wealth of the public in Nepal. Low vision is known to reduce vision-related quality of life (Rahi et al. 2009). Less than one-third of the participants were female. The finding of this study is similar to that of Shah and colleagues (2011). Future research in Nepal should identify whether fewer females have low vision or there are problems with accessing low-vision services. Our results also suggest that females get low-vision problems earlier than males: more than half of the females (58.97%) were in the age group 0–20 years but only one-third of the males (30.61%) were in this age group. It is a great challenge for our society that about two-thirds of the low-vision patients we saw were under 40.

## Visual acuity

**Figure 1.**
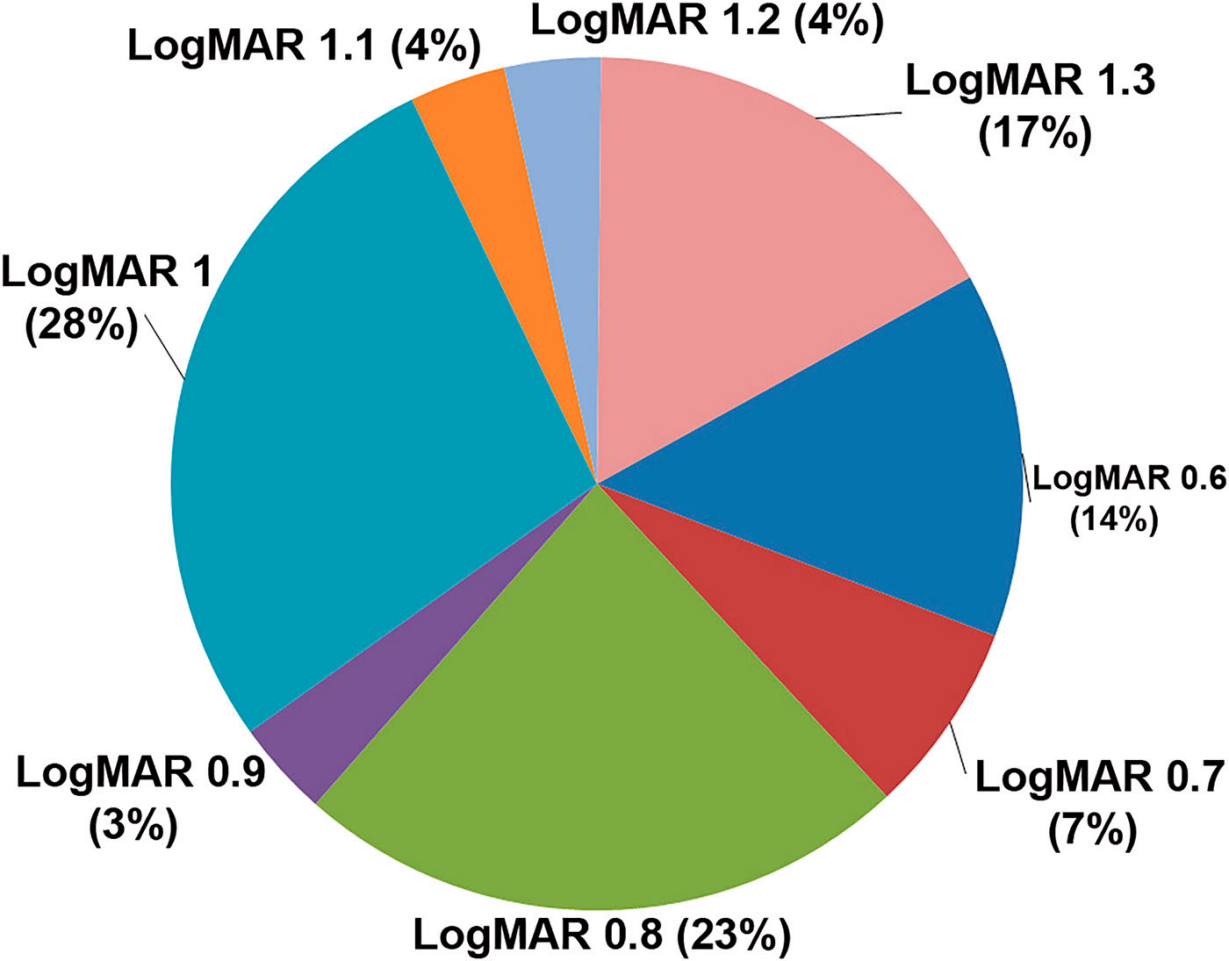
Best-corrected visual acuity of the participants. Overall, 78.10% patients had at least some amount of refractive error; 61 patients were myopic with mean age 34.85 years (sd: ±23.73). Their mean refractive error was −3.89D (sd: ±4.80D) with a range from −0.25D to −19.50D in the better eye. They had mean visual acuity of 0.92logMAR (sd: ±0.23). There were 46 hyperopic patients with mean refractive error +4.82D (sd: ±4.63D) in the better eye, with visual acuity of 0.91logMAR (sd: ±0.21).

Patients had mean visual acuity of 0.93logMAR (standard deviation: ±0.23) in the better eye with mode 1.00logMAR; 28% had visual acuity 1.0logMAR and 23% had 0.80logMAR (Figure 1).

Almost four-fifths of the low-vision patients seen had some amount of refractive error. Visual acuity can be improved by spectacle correction in many low-vision patients: for example, Sunness and El Annan (2010) have shown that refraction improves visual acuity by two lines or more in 11% of people with low vision (and by four or more lines in 3%). This highlights the importance of proper refractive correction for all low-vision patients.

## Cause of low vision

**Figure 2.**
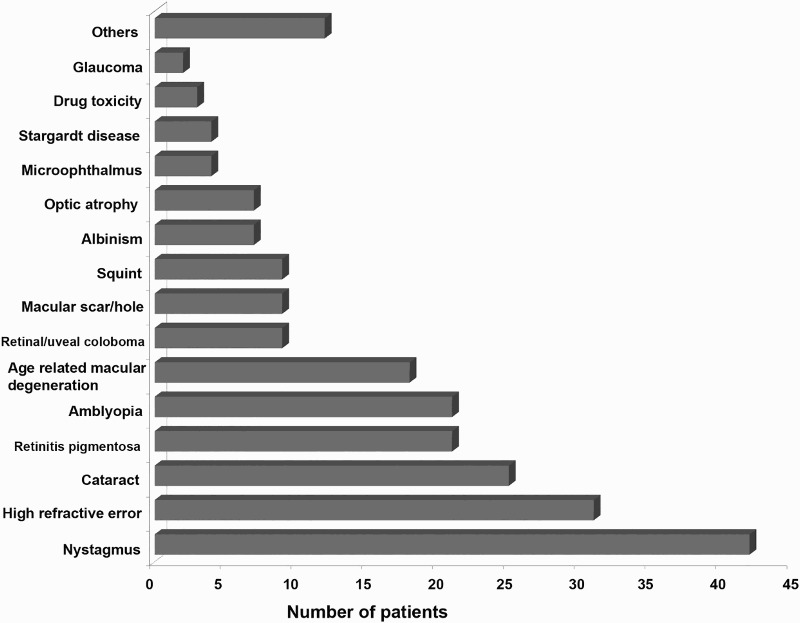
Causes of low vision. In the group of nystagmus patients, the mean age was 16.02 ± 7.08 years ranging from 4 to 29. Subjects with high refractive error (≥±5.00D) were older: mean age was 21.32 ± 14.38 years ranging from 5 to 77 in the group having high refractive error. There were 13 people with high myopia (9 male and 4 female) with mean myopia −11.54 ± 5.34D ranging from −5.25 to −19.50 in the better eye. Similarly, there were 18 people with high hypermetropia (15 male and 3 female) with mean hyperopia +9.85 ± 3.13D ranging from +5.00 to +16.00.

The cause of low vision was iatrogenic in 4 patients, 72 (52.6%) had 1 cause of visual impairment, 35 (25.5%) had 2 causes, 22 (16.1%) had 3 causes and 4 (2.9%) had 4 causes; 30.65% (42) of the participants had nystagmus, 22.62% (31) had high refractive error (≥±5.00D), 18.24% (25) had cataract. Retinitis pigmentosa, amblyopia and age related macular degeneration (ARMD) were found in 15.32%, 15.32% and 13.13%, respectively (Figure 2).

The mean age was 41.56 ± 28.79 years (ranging from 5 to 85) in the cataract group, and the mean age of the RP group was 38.9 ± 18.89 years (ranging from 11 to 70). The mean age of the amblyopic patients was 16.81 ± 11.01 years ranging from 4 to 56, and the mean age was 69.83 ± 12.23 years ranging from 42 to 85 in the patient group having ARMD.

Most of our patients had multiple causes of low vision. Nystagmus was found in highest number of patients (30.65%). Shah et al. (2011) also found nystagmus in highest number of patients (15%) in their study. Similarly, refractive error, cataract, retinitis pigmentosa, amblyopia and ARMD were found in higher number of cases in this study. However, Brilliant et al. (1985) found that cataract, retinal disease, trachoma, amblyopia and malnutrition were the main causes of blindness in Nepal. This may be due to the fact that this study population was highly dominated by lower age group. A recent survey (Thapa et al. 2011) done in Bhaktapur district of Nepal showed that the main causes of low vision were cataract, refractive error and glaucoma. Only 1.5% of people we saw had glaucoma as the cause of low vision. The different findings of this study may be due to the fact that the Bhaktapur study population was adult (age ≥ 40 years), but in our study, two-thirds of the participants were under 40. Moreover, our study was performed in a tertiary care centre and our population was the patients referred to the low-vision clinic by other ophthalmologists. In Nepal, many people are unaware of common eye diseases like glaucoma (Thapa et al. 2011). Others may not attend due to financial problems, transportation difficulties and the poor availability of eye care centre in many parts of the country.

In a European population, Buch et al. (2004) found myopia-related retinal disorders, diabetic retinopathy, cataract and retinitis pigmentosa to be the main causes of visual impairment, and ARMD as the major cause of blindness. Similarly, Nakamura et al. (2010) found that primary causes of low vision in Japan were cataract, corneal opacity, retinitis pigmentosa and diabetic maculopathy.

## Optical device of low vision

Glasses were prescribed for 107 (78.10%) patients with dioptric power ranging from +16 to −19.50. Telescopes were prescribed for 40 (29.2%) patients. Mean telescopic magnification was 3.58X (sd: ±0.71, range 2X to 5X). Handheld magnifiers were prescribed for 18 (13.1%) with mean power 3.06X (sd: ±1.66, range 2X to 7X). Closed circuit television and stand magnifier were each prescribed for a single patient. After conventional spectacles, telescopes were the most commonly prescribed optical low-vision device in this study. This is similar to the results of Kansakar et al. (2009) and to results from the USA (Scott et al. 1999), Holland (van Rens et al. 1991) and a university-based clinic in the UK (Leat & Rumney 1990). However, our results differ from studies based in British hospital (Lindsay et al. 2004; Crossland & Silver 2005) and Australia (Wolffsohn & Cochrane 1999) where the most frequently prescribed low-vision devices were non-illuminated hand magnifiers, illuminated hand magnifiers and illuminated stand magnifiers. These differences are likely to reflect the lower age of our clinic population, variation in the training of optometrists and differences in the availability of various low-vision aids.

## Conclusion

We report the patients seen and devices prescribed in a low-vision clinic based in a tertiary care centre in Nepal. Most of the low-vision patients seen were of working age, and about two-thirds were under 40. More than two-thirds of the low-vision patients were male, and female low-vision patients were younger than their male counterparts. Careful refraction and adequate optical correction reduce the burden of the visual impairment. Nystagmus, high refractive error, cataract, retinitis pigmentosa, amblyopia and ARMD were the main causes of low vision. Telescopes were the major optical devices prescribed for low-vision patients.
